# The uptake and effect of a mailed multi-modal colon cancer screening intervention: A pilot controlled trial

**DOI:** 10.1186/1748-5908-3-32

**Published:** 2008-06-02

**Authors:** Carmen L Lewis, Alison T Brenner, Jennifer M Griffith, Michael P Pignone

**Affiliations:** 1 Division of General Internal Medicine and Clinical Epidemiology, University of North Carolina, Chapel Hill, NC, USA; 2 Cecil G Sheps Center for Health Services Research, Chapel Hill, NC, USA

## Abstract

**Background:**

We sought to determine whether a multi-modal intervention, which included mailing a patient reminder with a colon cancer decision aid to patients and system changes allowing direct access to scheduling screening tests through standing orders, would be an effective and efficient means of promoting colon cancer screening in primary care practice.

**Methods:**

We conducted a controlled trial comparing the proportion of intervention patients who received colon cancer screening with wait list controls at one practice site. The intervention was a mailed package that included a letter from their primary care physician, a colon cancer screening decision aid, and instructions for obtaining each screening test without an office visit so that patients could access screening tests directly. Major outcomes were screening test completion and cost per additional patient screened.

**Results:**

In the intervention group, 15% (20/137) were screened versus 4% (4/100) in the control group (difference 11%; (95%; CI 3%;18% p = 0.01). The cost per additional patient screened was estimated to be $94.

**Conclusion:**

A multi-modal intervention, which included mailing a patient reminder with a colon cancer decision aid to patients and system changes allowing patients direct access to schedule screening tests, increased colon cancer screening test completion in a subset of patients within a single academic practice. Although the uptake of the decision aid was low, the cost was also modest, suggesting that this method could be a viable approach to colon cancer screening.

## Background

Colon cancer is the second leading cause of cancer-related deaths in the United States, and the third most commonly diagnosed cancer, with over 149,000 new diagnoses and 55,000 deaths expected in 2006 [1]. Colon cancer screening is effective in decreasing colon cancer incidence and mortality [2-4], and there are several recommended screening tests available to patients [5]. Despite its effectiveness, colon cancer screening is underutilized in the United States. Recent data on self-reported screening status from the Behavioral Risk Factor Surveillance System survey shows that only 57% of people in the United States are up to date with recommended screening [6].

Barriers at multiple levels of the healthcare system (physician, patient, and system levels) contribute to the underutilization of colon cancer screening, and targeting these barriers increases colon cancer screening [7,8]. One important barrier is lack of awareness about when screening is due. This barrier has been effectively targeted with system changes such as reminders to physicians or patients [9,10]. Another important patient barrier to screening is not understanding the importance of screening and difficulty choosing among multiple options. A colon cancer screening decision aid for patients has been shown to promote screening by educating and facilitating choice between different testing options [11]. However, implementation of decision aids in clinical practice can be difficult due to space and time constraints and may not reach all patients who are eligible; furthermore, patients who receive decision aids while in physicians' offices and are ready to be screened may still not have their preferred screening tests ordered by their physician [12].

Therefore, providing patients with decision aids outside of clinical practice, including information on how to obtain screening tests without an additional office visit, could produce a greater effect and be more efficient than providing decision aids in the practice setting. In this study, we sought to determine whether a multi-modal intervention, which included mailing a patient reminder with a colon cancer screening decision aid to patients and system changes allowing direct access to screening test scheduling, would be an effective and efficient means of promoting colon cancer screening in primary care practice. Primary outcomes were completion of colon cancer screening tests and cost of each additional patient screened.

## Methods

We conducted a controlled trial assessing the main outcome of colon cancer screening test completion for intervention patients and a waitlist control group at one practice site. Colon cancer screening receipt was determined by chart review five months after the intervention was mailed. The control group received the intervention materials after the study outcome data had been collected.

### Setting

We conducted this trial at the University of North Carolina Ambulatory Care Center in the General Internal Medicine practice (ACC-GIM). This is an academic practice that cares for over 5,000 adults aged 50 and older. The ACC GIM includes 15 attending physicians and 46 resident physicians.

### Patient Ascertainment

To track the status of colon cancer screening in patients at the ACC-GIM, we developed a tracking system in Microsoft Access. The database collects colon cancer screening test completion records from billing data obtained from the University of North Carolina (UNC) Healthcare System for flexible sigmoidoscopy and colonoscopy, as well as from lab results at ACC-GIM for fecal occult blood tests. Testing outside of the UNC System is uncommon but not captured in the billing database. Consequently, some patients who have no record of being up to date with screening may have, in fact, completed outside screening.

For this study, we used this database to identify patients ages 50 to 75 who did not have documented colon cancer screening in our database, *i.e.*, not having a colonoscopy in the last 10 years, flexible sigmoidoscopy in the last five years, or fecal occult blood testing in the last 11 months. We first identified 5,381 adults in this age range who were active patients, having been seen at the ACC-GIM at least once in the previous two years. We then identified 2,788 of the 5,381 (52%) who did not have documentation of being up to date with colon cancer screening. For this pilot study, we chose patients of attending physicians because we were more confident of the correct association in our database between patient and primary care physician than with patients of resident physicians. We identified 1,150 attending physician patients that met our eligibility criteria. Each attending physician reviewed their respective patient list and excluded patients who they deemed too ill to benefit from screening.

The remaining patients (n = 907) not excluded by the attending physicians were divided in half alphabetically by last name to form intervention and control/waitlist groups. A through L served as the pool for the intervention group and M through Z for the control/waitlist groups. From this pool, we selected a sub-sample to evaluate the intervention in depth prior to the full trial. We chose the first 137 patients of attending physicians listed alphabetically in the intervention pool for the intervention group and the first 100 patients of attending physicians in the control group.

### Description of Intervention

The intervention consisted of a mailed package containing the following materials:

• A letter, signed by the patient's physician, reminding the patient that they were due for screening and encouraging them to get screened.

• A survey entitled 'Colon Cancer Health Summary' to be completed prior to watching the video to determine screening history and personal or family history of polyps, colon cancer, or inflammatory bowel disease; from this information we could determine whether the patient believed that they were eligible for routine screening.

• A decision aid, 'Colon Cancer Screening: Deciding What's Right for You', in VHS and DVD format.

• A survey to be completed after watching the decision aid, measuring secondary outcomes of including interest and acceptability of the intervention.

• Information encouraging return of all materials, particularly the Colon Cancer Health Summary, regardless of screening status.

• Instructions and postage for returning the package.

The decision aid used in this study was created by the Foundation for Informed Medical Decision Making in conjunction with one of the authors (MP). The program is approximately 35 minutes long. A moderator leads a discussion about colon cancer and colon cancer screening. The first section describes colon cancer and the risk of getting colon cancer for those at average risk. The next sections of the decision aid video describe the different types of colon cancer screening tests, including: fecal occult blood test, sigmoidoscopy, a combination of fecal occult blood test and sigmoidoscopy, and colonoscopy. Each test was described in terms of how the test is completed, how often it needs to be completed, the amount of time needed to complete the test, effectiveness in finding polyps and cancer, convenience, discomfort, and risks associated with the test. Patient testimonials are interspersed for each testing option where patients describe their experiences with specific tests.

Detailed instructions on how to access the screening test of choice were included in the intervention package. For fecal occult blood testing, standing orders were implemented in the practice. A nurse facilitator was available by phone so patients could request fecal occult blood cards be sent to them and returned to the practice in a prepaid envelop. For flexible-sigmoidoscopy and colonoscopy patients were provided the number to the gastroenterology suite affiliated with UNC hospital. Schedulers in the gastroenterology suite were instructed to schedule patients who requested either test.

One month after the initial mailing, we sent a reminder letter to those intervention patients who had not returned materials. This reminder included a letter from the patient's physician stating again that they were due for screening, encouraging them to get screened, and reminding them to return the intervention materials.

### Primary Outcomes

#### Colon cancer screening test completion

Colon cancer screening test completion was determined by medical record review five months after mailing the intervention. The review was completed by two independent reviewers, one of whom was blinded to intervention status. Inter-rater reliability was assessed using the kappa statistic, and disagreements were resolved afterward by group discussion. The kappa statistic was 0.90 (95% CI 0.83, 0.96), indicating excellent agreement. The analysis was an intention to treat analysis. We included all patients in the intervention group even if they reported being at high risk or previously screened because we did not have similar information for the control group. This analysis allowed for the most conservative estimate of the intervention effect.

#### Cost

The other major outcome of interest was cost per additional patient screened. We calculated approximate cost per additional patient screened by estimating costs of postage, mailing materials, VHS and DVD duplication costs, and staff time spent sending the mailings (Table 1). We did not include staff time devoted to making the phone calls as this was considered part of the evaluation and not the intervention itself.

**Table 1:** Estimations for the cost per additional patient screened

	**Item Cost**	**Quantity**	**Cost**
Mail out postage	$1.84	137 mailed packages	$252.08
Mail back postage	$5.00	57 returned by patients	$285.00
Video/DVD duplication costs	$5.00	137	$685.00
Materials (Boxes and packaging	$1.00	137	$137.00
Staff time	$17.00/hour	9 hours staff time	$153.00
Total Cost			$1,512.08
Cost per additional patient screened	Difference in patients screened between intervention and control = 16 patients	$1,512.08/16	$94.51

#### Secondary Outcomes

For the intervention group, we attempted to determine several secondary outcomes, either through in the post-video questionnaire for patients who responded to the intervention by completing the written survey or via telephone interview for those that did not respond to the written questionnaire. Two months after the initial mailings, we attempted to contact via telephone those who either had not responded to the mailing (non-respondent) or those who had sent the materials back without a written response (non-participant). We made five call attempts to each non-respondent or non-participant. The intent of the calls was not to try to encourage screening, but to determine several secondary outcomes.

In both the post-video questionnaire and the telephone interview we asked questions about the acceptability of the intervention to those who were eligible for screening by asking if they liked receiving the decision aid in the mail. We also measured interest in screening and asked if they had attempted to schedule a screening test since receiving the intervention.

## Results

The intervention and control groups were similar in regards to age, sex, and race (Table 2).

**Table 2:** Patient characteristics in intervention and control group

	**Intervention**	**Control**
**N**	137	100
**Mean age**	62	62
**% Female**	60	61
**% White**	60	62
**% Black**	30	28

### Response to mailings

**Figure 1:**
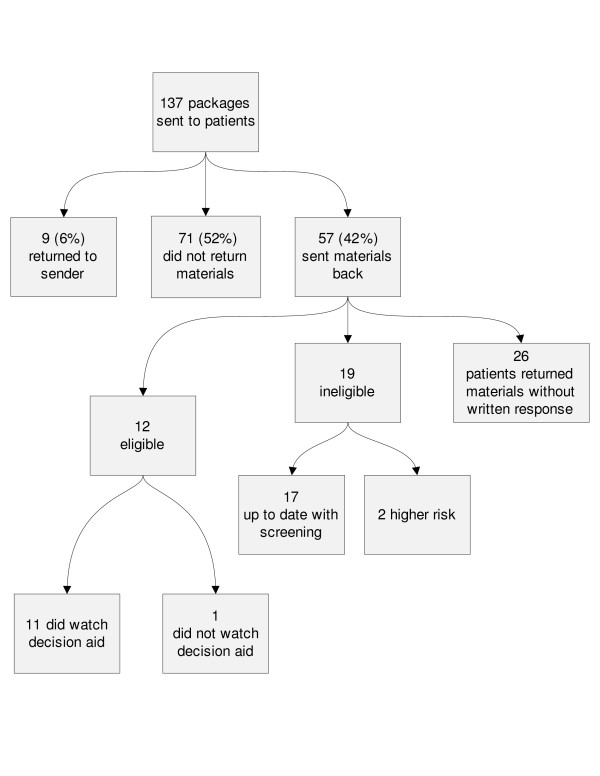
Response to mailing.

Of the 137 intervention patients, 57 people (42%) responded; 31 completed the materials, and 26 people did not complete any of the materials (non-participants). Among the 31 patients who completed materials, 12 reported that they were eligible for the decision aid, and 19 reported being up to date with screening or at high risk (Figure 1). Nine of the mailings were returned as undeliverable due to incorrect addresses (6%), and 71 patients (52%) did not respond.

### Primary Outcomes

#### Screening test completion

In the intervention group 15% of patients (20/137) were screened versus 4% of patients (4/100) in the control group (difference 11%;95% CI 3%, 18% P = 0.01

#### Cost per additional patient screened

The total cost of the intervention was calculated to be $1,512.08. The majority of the cost was attributed to the video costs ($685). The cost per additional patient screened was estimated to be $94 (Table 1).

### Secondary Outcomes

#### Responses to mailed survey

All eligible respondents (n = 12) reported that they were interested in screening; four had attempted to schedule a screening test since receiving the intervention materials and one reported difficulty in doing so. Eleven of the 12 respondents reported that they liked receiving the mailing and had watched the video. Five of these 12 obtained a screening test, four of whom had watched the video.

#### Responses to telephone interviews

**Figure 2:**
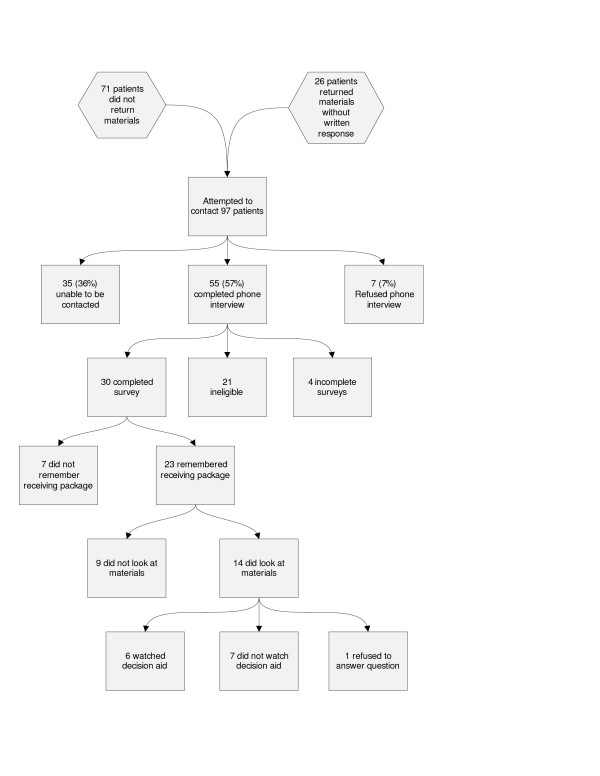
Phone call response.

We attempted to call both the 71 non-respondents and the 26 non-participants in the intervention group to determine how our program was received and their self-reported screening status (Figure 2). We were able to reach and interview 55 of these 97 people (57%). We found that 21 were either up to date with screening or at high risk. Of the remaining 30 people, 23 remembered receiving the package. Among these 23 patients, 14 reported that they had looked at the information, and six reported that they had watched the video. When asked about screening, 11 patients reported that they were interested in undergoing screening, and eight had tried to schedule a screening test. From the medical record review we found that three had obtained a screening test.

We asked those who had not watched the video why they had not watched it: 10 patients reported that they had not had time, two stated they did not have insurance so it was not worth it to them to watch the video, two reported that they were too scared of cancer to watch the video, one reported that they did not have either a DVD or VHS player, one had left the practice, and one did not provide an answer. We asked 15 of the 23 patients whether they liked receiving the decision aid; 12 liked receiving the package and three reported that they did not.

## Conclusion

We found that a multi-modal intervention, which included mailing a patient reminder with a colon cancer decision aid to patients of attending physicians and system changes allowing patients direct access to schedule screening tests, increased cancer screening test completion by 11% (95%; CI 3%;18% p = 0.01) when compared to a control group. We estimated the cost of the intervention to be $94 per additional patient screened. The response to the mailings was modest (42%), and about half who sent a mailed response did not complete the written materials.

Our multi-modal intervention included a reminder letter from their physician, a decision aid to educate and facilitate choice, and system changes to decrease barriers to colon cancer screening test ordering. In a recent review Stone *et al. *identified several studies that combined interventions in clinical practices to target colon cancer screening barriers at multiple levels [7]. However, none included a decision aid as a part of the intervention. We identified a randomized controlled trial performed by Zapka and colleagues that mailed a colon cancer screening educational video to 450 primary care patients before their scheduled appointment for a physical exam. Their study found no significant difference in screening rates between the intervention and control groups [13]. A potential reason for the discrepancy in findings with our study could be the intent of the video. The video used in our study was aimed at facilitating choice between screening options, whereas the video used in the Zapka *et al. *study was focused on increasing screening by flexible sigmoidoscopy. Additionally, our study provided direct access to screening tests, while the Zapka and colleagues' study encouraged discussion with providers and depended on the practice to arrange the screening appointment. Our previous work has shown that screening tests are often not ordered, despite patient interest [12].

Recent studies have used a similar mass mailing to patients to attempt to increase colon cancer screening rates with mixed results A study using self-reported screening completion as an outcome showed an increase in colon cancer screening of 23% over one year when fecal occult blood test cards and reminders were sent directly to patients [14]. Using claims data instead of self-reporting, Walsh and colleagues found no significant increase in colon cancer screening rates for colonoscopy and fecal occult blood tests after mailing patient educational materials and a letter encouraging screening; however, a small (3%) increase in flexible sigmoidoscopy was observed [15]. The 11% increase we found in screening rates could be attributable to the decision aid that differed from the educational materials sent by Walsh and colleagues, the system changes we implemented, or a combination of both.

Evaluating the costs of cancer screening programs is important in deciding which programs are most effective and efficient at promoting the targeted screening activity [16]. Most assessments of screening program costs have been conducted for breast cancer screening programs directed to patients [17]. The effectiveness and efficiency of mammography screening programs varies widely depending on the baseline screening rates, the target population, the intensity of the intervention, and the method for calculating costs. A low-intensity intervention similar to our study that included a tailored letter and telephone call to patients in a large HMO increased screening rates by 20%. This intervention had a relatively high estimated cost of additional patient screened of $818 [18]. Others have estimated costs for comparable programs to be much lower. Fishman *et al. *found that a telephone reminder increased screening by 16%, with an estimated cost of $92 for previously adherent women and $100 for those previously non-adherent [19]. Saywell targeted non-adherent women at a large HMO using telephone counseling and a physician reminder letter, which was shown to increase screening by 17% at an estimated a cost of $14 per additional patient screened [20]. Our estimated costs of $95 per additional patient screened compares favorably with the breast cancer screening programs because colon cancer screening may be less acceptable and is complicated by multiple testing options.

We were able to find only one other study that estimated the cost per additional patient screened for colon cancer screening. This study found comparable results to ours, but was aimed specifically at physicians. The intervention provided VA physicians with quarterly feedback on screening rates of their patients. This intervention increased colon cancer screening rates by 9%, with an associated cost per additional patient screened of $196 [17].

Our study has several important limitations. First, our sample size was small, making it possible that we found a difference in screening rates by chance. The relatively tight confidence intervals, however, suggest that this is unlikely. Second, the study included only one academic practice and a sub-sample of attending physicians' patients within the practice, limiting the generalizability of our findings. Third, the study was non-randomized, which could introduce bias if there were unmeasured differences between the intervention and control group patients. Because we had a limited number of patient characteristics available in our database we are not able to exclude differences between the groups as a possible cause for our findings. Although age, gender, and race were similar between the groups, insurance status was not available and could bias the control group to no screening if there were fewer insured patients in the control group. Although we found a difference in screening rates between intervention and control groups, we were unable to identify which part of the intervention was responsible for the difference in screening. Finally, our response rate to the initial mailings and calls was modest, despite five calls per patient. The views of the patients who refused or who we were unable to reach may differ significantly from those who we were able to interview.

A multi-modal intervention, which included mailing a patient reminder with a colon cancer decision aid to patients and system changes allowing patients direct access to schedule screening tests, was effective in increasing cancer screening test completion in a subset of patients within a single academic practice. Although the uptake of the decision aid was low, the cost was also modest, suggesting that this method could be a viable cancer screening program.

## Competing interests

The Foundation for Informed Medical Decision Making funded this study. The funding source had no role in the design, conduct, or reporting of the study or in the decision to submit the article for publication.

## Authors' contributions

All of the authors take responsibility for the findings reported in this work. Dr. CL drafted the manuscript. All authors participated in study design, analysis of data, interpretation of data, and revision of the manuscript. Furthermore, all authors have approved the manuscript in its final version
